# Estimation of carbon dioxide (CO_2_) reduction by utilization of algal biomass bioplastic in Malaysia using carbon emission pinch analysis (CEPA)

**DOI:** 10.1080/21655979.2020.1718471

**Published:** 2020-02-03

**Authors:** Nor-Insyirah Syahira Abdul-Latif, Mei Yin Ong, Saifuddin Nomanbhay, Bello Salman, Pau Loke Show

**Affiliations:** a Department of DVC Research, UNITEN R&D SDN BHD, Universiti Tenaga Nasional, Kajang, Malaysia; b Department of DVC Research, Institute of Sustainable Energy, Universiti Tenaga Nasional, Kajang, Malaysia; c Department of Chemical and Environment Engineering, Faculty of Science and Engineering, University of Nottingham Malaysia, Semenyih, Malaysia

**Keywords:** Plastic, CO_2_ emission, pinch analysis, algal biomass, bioplastic

## Abstract

Carbon dioxide (CO_2_) emission will increase due to the increasing global plastic demand. Statistical data shows that plastic production alone will contribute to at least 20% of the annual global carbon budget in the near future. Hence, several alternative methods are recommended to overcome this problem, such as bio-product synthesis. Algae consist of diverse species and have huge potential to be a promising biomass feedstock for a range of purposes, including bio-oil production. The convenient cultivation method of algae could be one of the main support for algal biomass utilization. The aim of this study is to forecast and outline the strategies in order to meet the future demand (year 2050) of plastic production and, at the same time, reduce CO_2_ emission by replacing the conventional plastic with bio-based plastic. In this paper, the analysis for 25%, 50% and 75% CO_2_ reduction has been done by using carbon emission pinch analysis. The strategies of biomass utilization in Malaysia are also enumerated in this study. This study suggested that the algal biomass found in Malaysia coastal areas should be utilized and cultivated on a larger scale in order to meet the increasing plastic demand and, at the same time, reduce carbon footprint. Some of the potential areas for macroalgae sea-farming cultivation in Sabah coastline (Malaysia), comprised of about 3885 km^2^ (388,500 ha) in total, have been highlighted. These potential areas have the potential to produce up to 14.5 million tonnes (Mt)/y of macroalgae in total, which can contribute 370 Mt of phenol for bioplastic production.

## Graphical Abstract

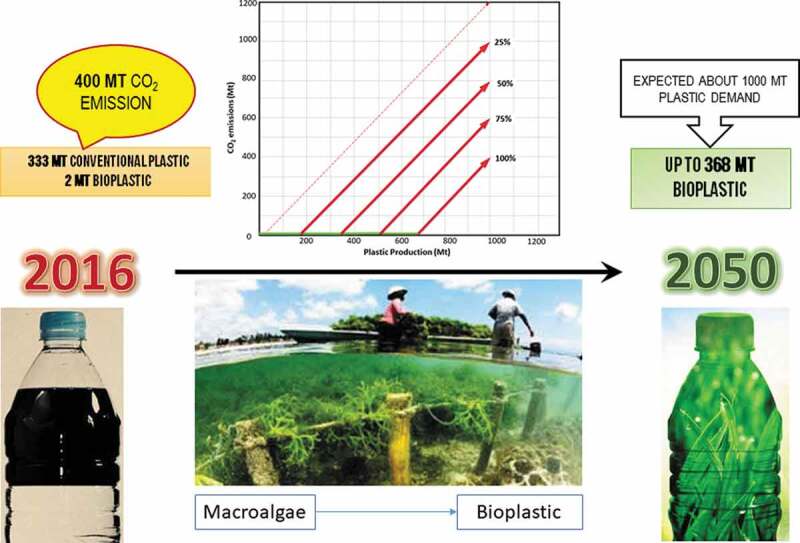

## 1. Introduction

A significant global issue regarding climate change and greenhouse gas (GHG) emission (primarily carbon dioxide – CO_2_) has been highlighted in the past few years. Human activities, such as burning fossil fuels for electricity, heat and transportation purposes [1], are the main contributors to the increasing GHG emission. Moreover, this issue is further promoted by the rapid fossil fuel–dominated urbanization and industrialization process since 2000 [2,3]. Carbon emissions from the global energy industry rose at the fastest rate in almost a decade, after extreme weather and surprise swings in global temperatures are stoked by extra demand for fossil fuels [4]. In conjunction with that, many parties are aware of the necessity of reducing the reliance on petroleum-based oil that has been exploited for several purposes. Currently, many concerns have focussed on the energy, including electricity and transportation that cause a huge negative impact on carbon emission [5,6]. However, there is still a lack of concern on petroleum-based plastic production even though it contributes only a small portion, around 1% of the total global carbon emission [7,8]. Furthermore, there are limited studies done to track the growing usage of bioplastic with the purpose of carbon emission reduction.

Plastics have outgrown most man-made materials since the 1950s due to its versatility and functionality. However, most of the plastic is non-biodegradable and hence causing environmental problems. Concern about plastic pollution has led to the act restrictions on certain plastic production and promotes the replacement of other types of plastics [9]. Some of the countries implemented plastic ban, including Australia and Malaysia [10–12]. The Malaysian government also promotes bioplastics and encourages eco-friendly products to substitute single-use plastics as part of its efforts to move toward a more sustainable environment [11]. Non-government environmental groups have also reacted to the government’s decision to extend the 20-sen plastic bag charge to all types of business premises in 2022, by calling for a complete ban on the use of plastic bags [12].

Conventional plastics are mostly made of heavy crude oil, and so their production is highly related to the fossil resource depletion and climate change issues [13]. Besides concern on the waste management problem of the conventional plastic with low degradability, the GHG emission is also another main concern. Thus, the increasing trend of global plastic production generates a significant negative effect on the environment [14]. Plastics are organic materials that can be either fossil fuel–based or bio-based. Both types of plastic materials are recyclable, and it is possible to produce biodegradable plastics with both types of feedstocks. Bioplastic materials and plastic products are extremely resource efficient along their service life, helping us to avoid food waste, to save energy and to decrease CO_2_ emissions [15,16]. Hence, instead of finding alternative ways to reduce the global demand for plastic, another option is to increase the productivity in a more reliable and sustainable way. Many parties and authorities nowadays concern and strive to replace conventional plastic production with bioplastic, which can be acquired from numerous types of biomass. Bioplastic production capacities are estimated to grow by more than 400%, with Asia (including Thailand, India and China) expanding its role as a major production hub, according to the figures released at the 9th European Bioplastics conference [17].

In the current view, the bioplastic resin can be derived from agricultural waste, palm oil, corn, sugarcane and some other biomasses, considering their high availability and crop production [18,19]. Bioplastics have been increasingly spotlighted as one of the effective means to save fossil fuels, reduce CO_2_ emission and overcome plastic waste management. The biodegradability of bioplastics has been widely publicized in society, and the demand for bioplastic packaging is also rapidly increasing among the retailers and food industry on a large scale [7,20]. Besides, seaweed and microalgae have been considered as a potential feedstock for bio-based plastics due to their high growth rate and extensive environmental tolerance [21].

There are two types of algae, namely microalgae and macroalgae (also known as seaweed). It is already well known that microalgae have higher lipid content compared to macroalgae, but their cultivation and harvesting is more difficult [22]. This study will focus more on the utilization of macroalgae or seaweed, as an alternative to produce a larger quantity of bioplastic. Algae species can grow and survive over a wide range of temperatures and conditions. Malaysia is located within the area which has a huge potential to maximize algae productivity. Geographically, the South China Sea border Peninsular Malaysia in the east and both Sarawak and Sabah in the north have natural advantages for algae culture. Malaysia has various salt lakes that offer researchers to develop algae-based technology. Hence, Malaysia is surrounded by sea and has an extensive coastline fringed by numerous islands, providing various habitats for the proliferation of tropical algae [7]. In this analysis, some of the potential areas for further seaweed cultivation were determined in order to meet the target of plastic production, in view of reducing the possible CO_2_ emission in the future.

Process integration methodologies, such as pinch analysis or mathematical programming, have been successful in providing answers and support for essential economic development, better utilization and savings schemes, and have been widely used [23]. The focus of this paper is to make a general forecasting study on the relation of global plastic production with CO_2_ emission by 2050 using carbon emission pinch analysis (CEPA). CEPA is commonly implemented for several established studies as a methodology to estimate the potential carbon reduction from energy sector. However, in this study, CEPA is used to predict the carbon reduction from global plastic production. The effect of global plastic production is always being ignored as it has a minor contribution to total global carbon emission. In this study, CO_2_ emission, specifically from plastic production, from the year 2016 to the year 2050, has been predetermined and identified in order to estimate the potential carbon emission reduction. To achieve the predicted carbon reduction, the required amount of bioplastic in 2050 is also highlighted in this paper.

## 2. Methodology

**Figure 1.**
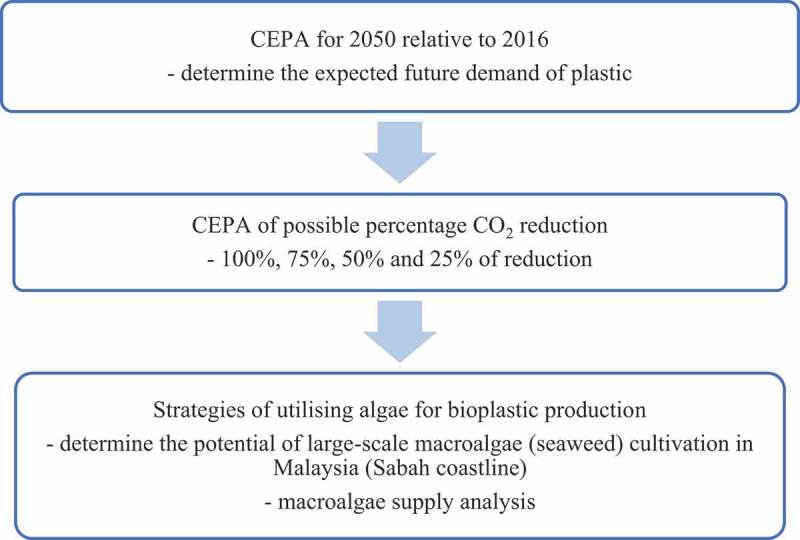
Process flow of methodology.

An interdependence research approach was implemented in this study. Figure 1 shows the process flow of methodology for this particular study.

CEPA was used to determine the relationship between global plastic production (including bioplastic) and their corresponding CO_2_ emission levels in the view of the year 2016. The average emission factor was then predetermined, and the forecasting study was carried out to predict the total demand for plastic in the year 2050, based on the current trend. Hence, with the available preliminary data, further pinch analysis was carried out for the determination of the total needs of bioplastic production in 2050 to replace conventional petroleum-based plastic material in order to reduce the potential carbon emission prediction. The analysis considers the possible reduction of CO_2_ emission from plastic production based on 100%, 75%, 50% and 25% reduction.

The possible strategies for macroalgae utilization within Malaysia were then specified in this study according to preliminary value from CEPA analysis. Then, some possible value of algae production was determined by utilizing the potential areas in Sabah and land-based cultivation requirements in order to fulfill the targeted algal biomass. The potential bioplastic products from algae utilization strategies are then calculated and analyzed. In order to analyze and estimate the total potential area for algae cultivation, Google Maps Area Calculator online tool/software (Daft Logic) was used [25,26].

The oil content of macroalgae is generally less than microalgae. However, Pohl and Zurheide reported that lipids of some macroalgae (seaweeds) were reported to be very high, up to 51% of the total fatty acids. From the previous study, it is reported that *Chaetomorpha linum* contains 15% of oil [27]. Typically, liquid yields of 60–75 wt%, char yields of 15–25 wt% and non-condensable gas yields of 10–15 wt% are observed in macroalgae [28]. In previous sentence, it is mentioned that liquid yields around 60–75 wt%, based on previous researches (with reference). Hence, the assumption made for this particular study, based on the maximum value, which is 75% liquid yield.

Equation:

(1) P=QM ×β×μ,

where *P* is the potential phenol from feedstock in tonne/year, *QM* is the quantity of biomass in t/y, *β* is conversion efficiency and *µ* is theoretical liquid yield obtained from pyrolysis.

Some general assumptions have been made:

- 100% reduction: CO_2_ emission is maintained at current CO_2_ emission from plastic production, which is about 400 Mt CO_2_ emission level although the production of plastic is increasing
- 10% of bioplastic is derived from other biomass, 90% target utilization from algae
- Theoretical liquid yield from pyrolysis = 0.75
- Theoretical conversion efficiency = 0.99

## 3. Results and discussions

### 3.1. Carbon emission pinch analysis

A global statistic depicts the global plastic production from 1950 to 2016. It is reported that in 2016, world plastic production totals around 335 million tonnes (Mt) [15]. With the increasing demand for plastic, carbon emission is also expected to increase. Indeed, it is estimated that 90% of plastics are produced from fossil fuel feedstock, and the production gives rise to approximately 400 million tonnes of GHG emissions (primarily CO_2_ per year globally) [13]. Worldwide, plastic production has been growing exponentially and could reach up to 1.2 billion tonnes annually in 2050. In 2016, plastic production was responsible for 1% of global carbon emission and it is expected to be 3% by 2050.

Table 1 shows an established data that can be used as an indication for pinch analysis. The aim of this pinch analysis is to maintain 1% of carbon emission until the year 2050, which is 400 Mt. The assumption that biomass-based material or bioplastic contributes to zero carbon emission was considered.

**Figure 2.**
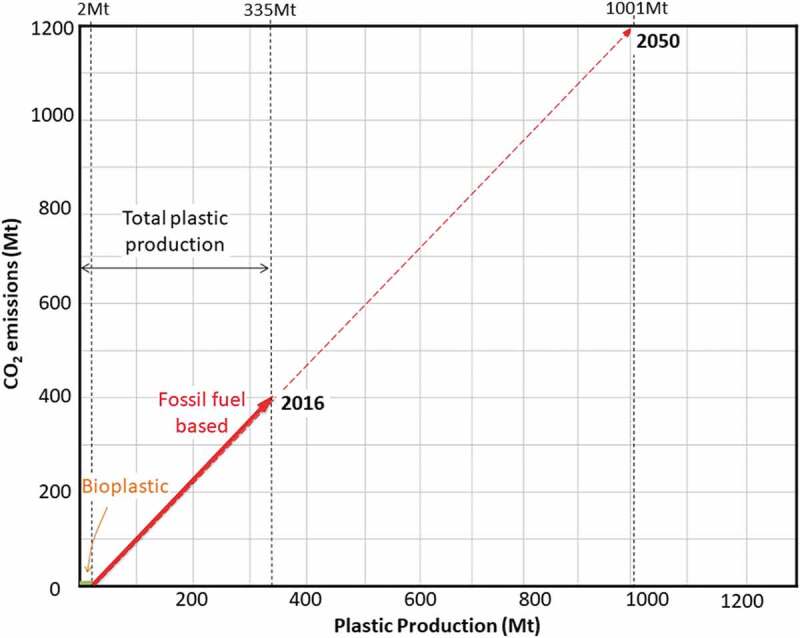
Projection to 2050 of plastic production.

**Table 1.** Global greenhouse gas emission in 2016 and 2050.

Year	Plastic production (Mt) [15]		Greenhouse/CO_2_ emission (Mt)	Percentage of total emission
2016	335	333	400	1%
		(conventional plastic)		
		2		
		(bioplastic)		
2050	Prediction from pinch analysis		1200	3%

The survey on the current trend of global plastic production shows that CO_2_ emission will surely increase in the future. Thus, this study was performed to predict the plastic production and CO_2_ emission in 2050, relative to 2016, as an indication for the current trend. Referring to Figure 2, plastic production in 2016 was about 335 Mt, including the conventional fossil fuel–based plastic and also bioplastic [15]. It is also estimated that in that particular year, the production of bioplastic, mainly from agricultural food waste, was around 2 Mt [29]. It is also reported that 400 Mt of CO_2_ was emitted by plastic production alone in 2016. As bioplastic is assumed to have zero CO_2_ emission, the total 400 Mt of emission is contributed by petroleum-based plastic production, about 333 Mt on average. Then, the emission factor can be estimated as about 1.2 for conventional plastic production. Hence, for this analysis, it is assumed that there will be about 1.2 Bt of CO_2_ emission, with 1 Bt total plastic production demand, in 2050 if the supply of plastic still relies on fossil fuel.

**Figure 3.**
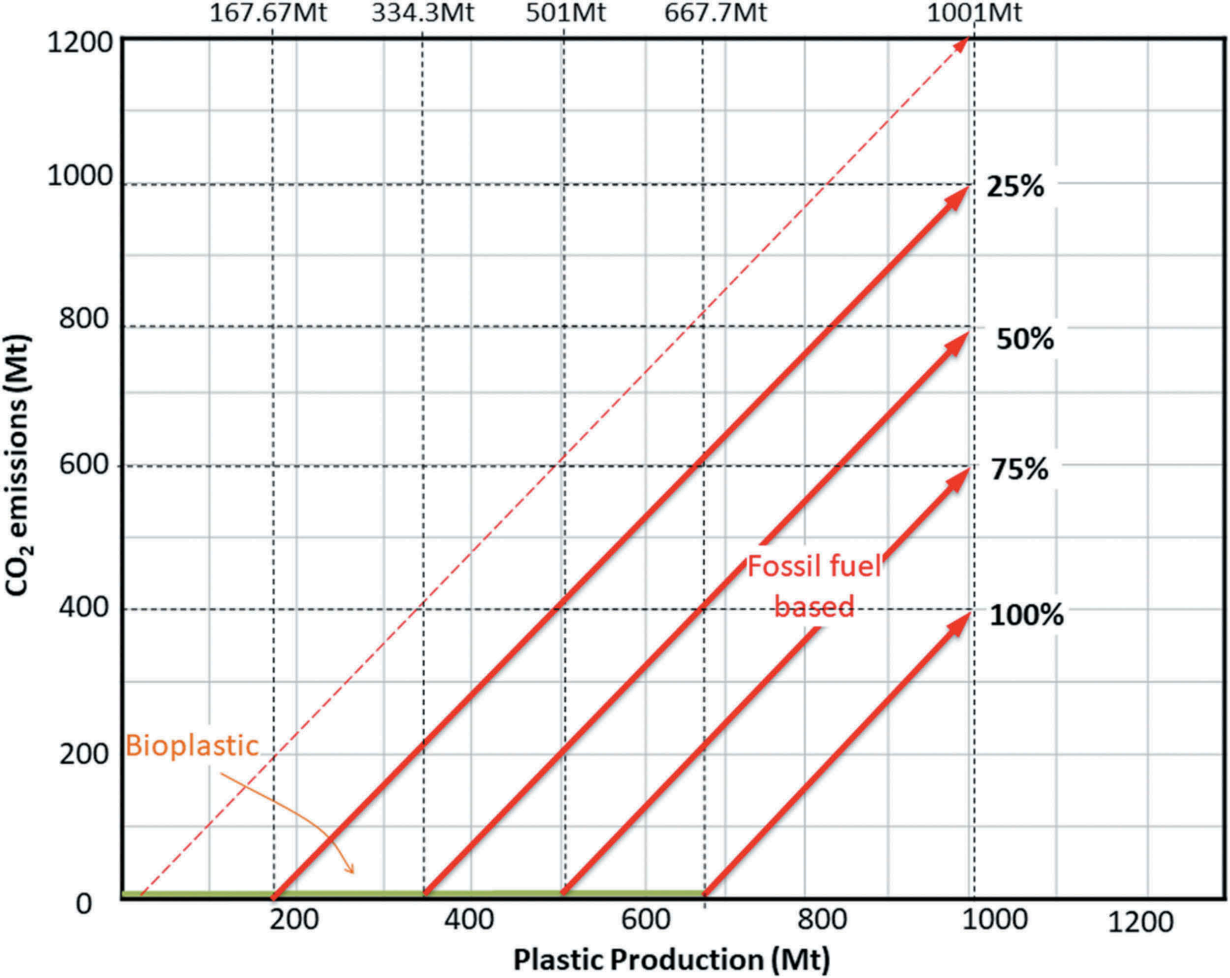
Carbon emission pinch analysis (CEPA) by percentage reduction.

As mentioned previously, with the current trend of plastic production, the carbon emission in 2050 is expected to achieve about 1.2 Bt (1200 Mt) of emission. Hence, the estimated total demand for plastic production in 2050 is around 1 Bt. Based on the targeted results of CEPA, the renewable bioplastic production target was evaluated. Based on the current status, 2 Mt of bioplastic production is mainly produced from agricultural food waste and palm oil. It is believed that more potential biomass can be introduced as a potential feedstock to be converted into bio-oil with optimum phenol content for plastic production.

Figure 3 is another pinch analysis, which shows the reduction of CO_2_ emission that can be achieved by increasing the biomass supply for plastic production. The green line indicates the target of plastic being produced by bio-based material. The intersection between the green flat line and the shifted gradient line shows the amount of plastic that is needed to be replaced with bio-based material in order to reduce the CO_2_ emission at respective percentages, in projection to 2050.

To maintain the CO_2_ emission at 400 Mt (the current CO_2_ emission amount), the amount of plastic production, that is suggested to be substituted with zero emission sources, needs to be evaluated in order to meet the estimated demand in 2050 as stated previously. From the pinch analysis, it is observed that about 668 Mt bio-based plastic should be produced as an alternative to conventional fossil fuel plastic, while the remaining 333 Mt can be produced conventionally. However, the results show that bio-based plastic production needs to be twice higher than conventional plastic.

Hence, this analysis was re-conducted by considering at least 25% of reduction from the expected amount of emission, resulting in about 167 Mt of bioplastic that need to be produced in order to meet the target demand. In other words, there are about 133.5 Mt algae needed to be cultivated, purposely for bioplastic production. For macroalgae to be in a position to supply even a small portion of this bioplastic need, it is apparent that it needs to be cultivated on very large scales. Assuming 75% liquid yield per hectare per year and 99% conversion efficiency, the potential of cultivating the algae on a large scale is generally figured out in this paper.

Table 2 summarizes the data from the graphical pinch analysis (Figure 3), showing the amount of bioplastic required as an alternative to reduce the global CO_2_ emission from plastic production. In order to maintain CO_2_ emission at 400 Mt in 2050 (100% CO_2_ reduction), bioplastic production should be more than 600 Mt to replace the plastic derived from fossil fuel–based or petroleum-based, which is a very large amount compared to the current production. The target of CO_2_ reduced by 25%, 50% and 75% is also represented in the graph above.

**Table 2.** Carbon reduction and the expected bioplastic production demand.

Percentage reduction	100%	75%	50%	25%
CO_2_ emission (Mt)	400	600	800	1000
Fossil fuel–based plastic (Mt)	334	500	656	834
Bioplastic (Mt)	667	501	345	167

### 3.2. Bioplastic utilization opportunity

**Figure 4.**
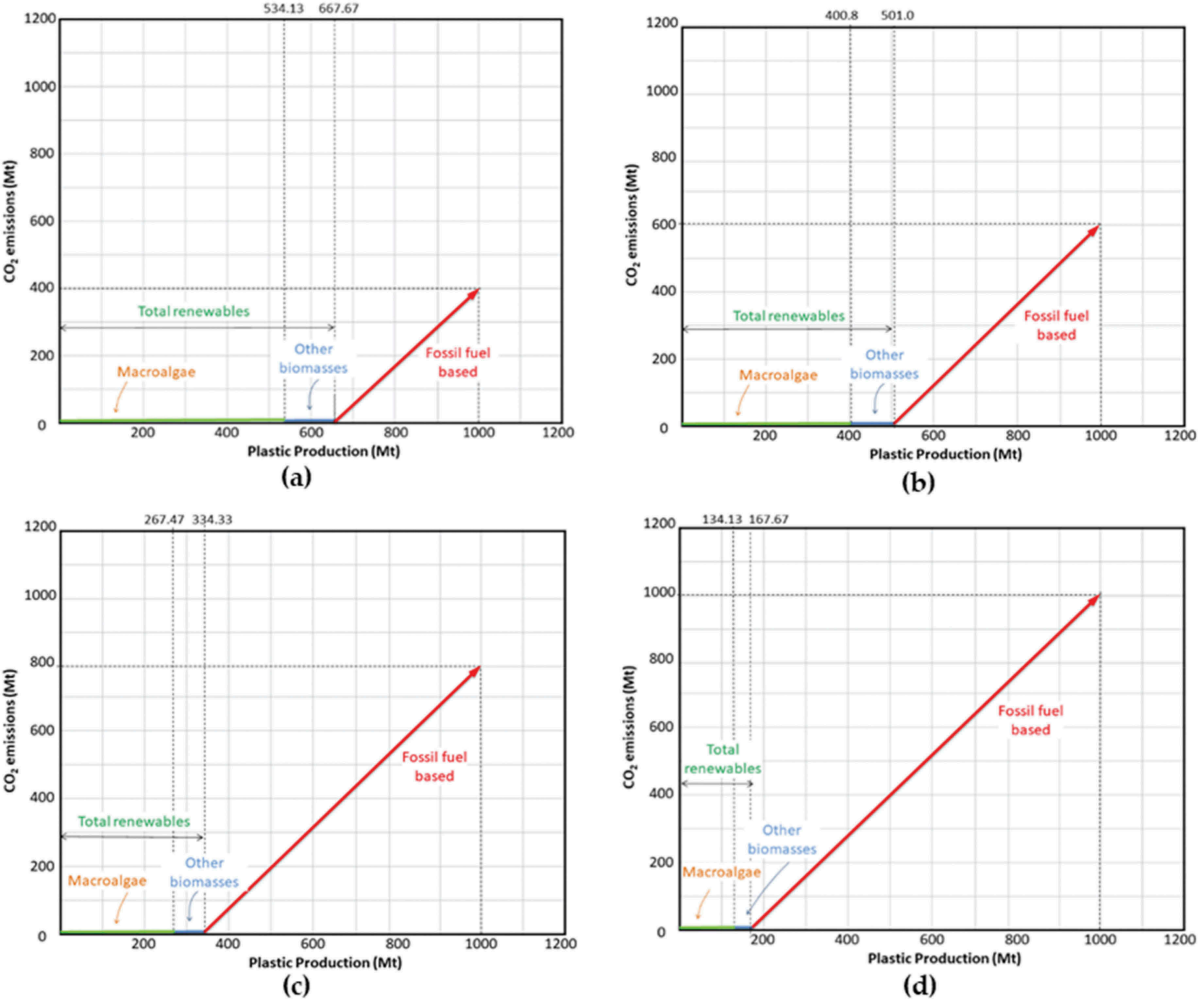
Carbon emission pinch analysis (CEPA) for (a) 100%; (b) 75%; (c) 50%; (d) 25% CO_2_ emission reduction.

The percentage of CO_2_ reduction is calculated using the CEPA, as mentioned in the previous section. Figure 4(a-d) shows the pinch analysis for specified percentage reduction. Figure 4(d) shows the pinch analysis for 25% CO_2_ reduction, which is the minimum target percentage for this particular study, while Figure 4(a), Figure 4(b) and Figure 4(c) represent the pinch analysis for 100%, 75% and 50% CO_2_ reduction, respectively. By considering the remaining conventional fossil fuel–based plastic, it is almost impossible to completely eliminate CO_2_ emission. A 100% CO_2_ reduction, in this case, means that the CO_2_ emission is maintained at about 400 Mt CO_2_ emission level (current CO_2_ emission from plastic production) although the production of plastic is increasing based on the expected demand.

In future view, plastic products may be produced from many raw materials including fossil fuel and different types of biomass. There are many other alternatives and improvements have been explored for bioplastic production [15,20,30]. The data of expected plastic production are presumed and shown in Table 3.

**Table 3.** Carbon reduction and the expected bioplastic production demand from macroalgae.

Percentage reduction	100%	75%	50%	25%
CO_2_ emission (Mt)	400	600	800	1000
Fossil fuel–based (Mt)	333.33	500	666.67	833.33
Bioplastic				
Algae biomass (Mt)	534.13	400.80	267.47	134.13
Other biomass (Mt) (20% of bioplastic)	133.53	100.2	66.87	33.53
Annual phenol demand, P (Mt/y)	15.71	11.79	7.87	3.95
Annual demand quantity for algal biomass, QM (Mt/y)	21.16	15.88	10.59	5.31

From the theoretical data, about 83 Mt of bioplastic resin from algae need to be produced. In order to produce 83 Mt in coming 34 years, 2.5 Mt of algae need to be produced every year in relation to 2050 demand. By referring to the equation above, the quantity of raw biomass in t/y is determined, resulting in about 3.3Mt of algae that need to be produced every year.

### 3.3. Macroalgae development strategies

Algae have been traditionally cultivated in Asia for centuries, especially in China and in Indonesia. Macroalgae (seaweed) have diverse species which can be produced either by cultivation or by harvesting from wild [31]. There are many different technologies available to produce macroalgae (seaweed), but optimization and more efficient developments are still required. Two main established options of seaweed cultivation development are sea farm and tank culture system. Seaweed farming is one of the top priorities set for development in Malaysia due to the increasing world demand for processed seaweed. Seaweed farming has been identified as one of the high-impact aquaculture activities in Malaysia due to the increasing world demand for raw and processed seaweed, which was about 350,000–400,000 tonnes in 2012 [30].

#### 3.3.1. Utilizing the crops for seaweed production and cultivation on potential area

**Figure 5.**
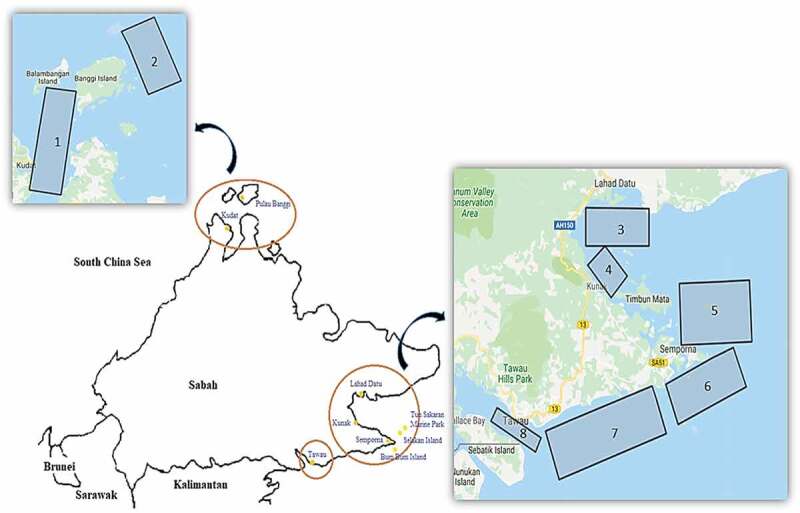
Potential area for large-scale macroalgae (seaweed) cultivation in Sabah, Malaysia.

Sabah is the main seaweed producer in Malaysia and most of the total production is farmed off in the east coast of Sabah. The main seaweed cultivation sites in Sabah are located in the east coast of Sabah, such as Semporna, Kunak and Lahad Datu. Cultivation of seaweed in other coastal districts in the west coast of Sabah has been developed slowly [32]. Google Maps Area Calculator online tool/software (Daft Logic) was used to analyze and estimate the total potential area for algae cultivation, as stated in Section 2. Based on the data of the potential area from previous research [7,33], the areas are specified as shown in Figure 5.

Semporna is already known as the main seaweed producer in Sabah, and it is believed that there are many more unutilized areas for harvesting seaweed. Several potential areas have been determined and specified in Figure 5, and the total calculated area for a specified location for macroalgae (seaweed) cultivation in Sabah coastline is shown in Table 4.

**Table 4.** Total area for large-scale macroalgae (seaweed) cultivation in Sabah.

Location [33]	Area	Potential area (km^2^)	Potential area (ha)
Kudat	1	820	82,000
Pulau Banggi	2	610	61,000
Lahad datu	3	430	43,000
Kunak	4	180	18,000
Tun Sakaran	5	78	7800
Selakan Island	5 & 6		
Semporna	6 & 7	575	57,500
Bum bum Island	6		
Semporna-Tawau	7	1050	105,000
Tawau	8	140	14,000
**Total potential area**		**3885**	**388,500 ha**

Large-scale algae cultivation has been implemented in countries such as Japan, Korea, Taiwan and China, which have cultivated macroalgae on areas of about 80,000 ha in total. These possibly represent the largest scales on which macroalgae have been cultivated in an organized manner by the industry – most other countries have cultivated macroalgae on much smaller scales (Vietnam, for instance, is reported to have about 5,000 ha under cultivation for macroalgae) [13]. Thus, even though the cultivation of macroalgae on millions of hectares does not appear infeasible, currently there is no adequate understanding of all the factors that could affect such feasibility. It is, however, likely that many of the processes and methods that are being used to cultivate algae by those established countries could be employed in Malaysia in the future.

Obtaining reliable data on biomass yields is difficult, as most macroalgae are harvested from wild populations. While there is a large amount of reported productivity data for a range of seaweed species worldwide, making generalizations is still quite difficult owing to a number of reasons [27]. However, the average global yield of seaweed can range from 12 to 60 t/ha. Hence, by considering the seasonal factor, the average yield obtained is assumed as 36 t/ha in this analysis for sea-farming implementation. From the data of the area obtained, the total potential amount of algae from sea farming from utilization of 388,500 ha is about 13.986 Mt every year by effective seaweed production annually until 2050. With the increasing concerns on algal biomass productivity, the technology of algae cultivation is expected to emerge gradually from time to time and improve the production yield, which can even double up.

Presently, seaweed cultivation takes place in more than 50 countries worldwide, and there are more latent areas that can potentially be utilized for macroalgae (seaweed) cultivation. For instance, Indonesia, as an archipelago with 17.504 islands and has long reach 81.000 km coastline, has huge potential for the development of seaweed, where development activities have been carried out in the marine areas of Indonesia from Nanggroe Aceh Darussalam to Papua [34]. It shows a huge potential of cultivating macroalgae, particularly in Asia, as an alternative strategy to be employed for algal bioplastic production.

#### 3.3.2. Self-cultivation (land-based cultivation)

Based on their mode of cultivation, seaweeds are classified as wild seaweed and aqua-cultured seaweed. About 1 million tonnes of macroalgae are harvested annually from natural stocks. For some macroalgae like the drift seaweeds, location and seasonal availability are unpredictable. Hence, land-based seaweed cultivation is usually preferred where they can be cultured either in ponds or in tanks. Land-based cultivation varied in a wide range. One of the most established systems is tank culture system. The use of tanks may provide the greatest productivity per unit area per day and is more efficient than any other type of farming as they can provide the necessary aeration and nutrients to the growing algae under controlled conditions. Ponds are larger, and hence it might prove to be difficult to grow the seaweeds under control [27]. Meanwhile, aqua-culturists prefer growing macroalgae in the sea.

Considering maximum yield can be achieved which is 60 t/ha by land-based cultivation, large-scale seaweed cultivation on the land can give a significant contribution to seaweed resources. For this analysis, assuming 10,000 ha of land space is allocated for seaweed cultivation, it can grant up to 0.6 Mt annual production of macroalgae in 2050.

#### 3.3.3. Macroalgae supply analysis

Considering both the methods of large-scale cultivation for meeting the phenol demand, the analysis data are outlined as shown in Table 5.

**Table 5.** Large-scale cultivation method and expected contribution for bioplastic production.

Large-scale cultivation method	Expected yield (t/ha)	Expected annual seaweed production QM (Mt/y)	Expected annual phenol production P(Mt/y)	Expected total quantity of seaweed can be produced in 2050 (Mt)	Expected total phenol can be produced in 2050 (Mt)
Sea farming	36	13.986	10.385	475.524	353.077
Self-cultivation	60	0.6	0.446	20.4	15.147
Total		14.586	10.831	495.924	368.224

In this study, the authors focussed more on sea farming becauseit is easier to control. Moreover, the sea-farmingcultivation in Sabahalready existed in large scalea and is suggested to be up-scaled. Meanwhile, the large-scale land-based cultivation needs more systematic control and needs to be initiated properly in the long run. From the data in Table 5, it is estimated that 13.986 Mt/y of seaweed can be produced from sea farming, while about 0.6 Mt/y from self-cultivation. As a result, it is expected that about 368 Mt of total phenol can be utilized for plastic production.

## 4. Conclusions

GHG and CO_2_ emissions from global plastic production are expected to be increased with the increasing demand for plastic. In order to maintain the global CO_2_ emission in 2050, CEPA was conducted and general strategies of utilizing algae for bioplastic in order to replace conventional fossil fuel–based plastic have been outlined. This forecasting study considered the global issue, but the general strategy analysis was done within the scope of Malaysia region only. In order to replace the expected usage of conventional plastic with bioplastic, large-scale cultivation is required for macroalgae, and hence, the potential area for algae cultivation was determined. From the analysis, by considering the average yield for the crop, the focussed area around Sabah coastline, which is about 3885 km^2^ (388,500 ha), can potentially produce up to 13.986 Mt/y of seaweed in 2050. From the total expected macroalgae/seaweed supply, including self-cultivation which is less than 1 Mt/y, the total phenol that can be utilized for bioplastic production is about 370 Mt. By considering the required amount of algae-based bioplastic in 2050, in order to achieve 100% CO_2_ reduction, an area of more than 0.6 million hectares is needed for macroalgae cultivation for bioplastic production.

## Research highlights

- Estimating CO_2_ emission from expected plastic demand in 2050, using CEPA analysis.
- Algal bioplastic is suggested to replace the conventional fossil fuel–based plastic.
- The potential of large-scale seaweed cultivation at Sabah to support algal bioplastic.
- Analyze the potential of CO_2_ reduction by algae utilization for bioplastic synthesis.
